# Supplementary material for: A high-resolution mRNA expression time course of embryonic development in zebrafish
Source: eLife. 2017 Nov 16;6:e30860. doi: 10.7554/eLife.30860 (PMC5690287; doi:10.7554/eLife.30860)
Supplement: Supplementary file 6. [file elife-30860-supp6.zip › biolayout-clusters-files/Cluster179-genes.html]

Cluster179


# Cluster179: Genes

| Ensembl ID | Gene Name | Chr | Start | End | Biotype |
| --- | --- | --- | --- | --- | --- |
| ENSDARG00000079046 | RABL6 (1 of many) | 5 | 5120198 | 5182360 | protein\_coding |
| ENSDARG00000032072 | cdk16 | 8 | 8323856 | 8408083 | protein\_coding |
| ENSDARG00000089930 | iqce | 1 | 11167910 | 11192045 | protein\_coding |
| ENSDARG00000025859 | lmf2b | 4 | 9477584 | 9501930 | protein\_coding |
| ENSDARG00000010400 | lrrfip2 | 13 | 42495145 | 42547945 | protein\_coding |
| ENSDARG00000086927 | pik3c2b | 11 | 23354386 | 23458894 | protein\_coding |
| ENSDARG00000061952 | si:dkey-245p14.7 | 1 | 18869815 | 18906338 | protein\_coding |
| ENSDARG00000006712 | wipf2b | 19 | 5685989 | 5703046 | protein\_coding |
